# Influence of Information Sources on Women’s Behavioural Practice towards Breast Cancer in Lagos, Nigeria

**DOI:** 10.3390/healthcare10101901

**Published:** 2022-09-28

**Authors:** Precious Adedoyin, Evaristus Adesina, Babatunde Adeyeye, Kehinde Oyesomi, Hezekiah Falola

**Affiliations:** 1 Department of Mass Communication, Covenant University, Ota 112233, Nigeria; 2 Department of Business Administration, Covenant University, Ota 112233, Nigeria

**Keywords:** breast cancer, breast self-examination, information sources, health belief model

## Abstract

The global burden of breast cancer is increasing with an effect on the physical, mental and socio-economic wellbeing of the human population. Existing studies have majorly focused on the prevalent rate and clinical analysis of the disease, to the neglect of the nexus of information sources and breast cancer behaviours. This study examined the influence of information sources on perceived susceptibility, perceived severity and perceived barrier of women towards breast cancer and breast self-examination in Lagos state, Nigeria. A cross-sectional survey was employed in eliciting information from 400 women respondents randomly selected through the multistage sampling technique method. The study revealed that Internet-related channels of communication had a greater influence on susceptibility and severity perceptions of women. On the perceived barrier of women towards breast self-examination, the majority were influenced by interpersonal networks of communication (friends and relatives). Based on the findings, this study concludes that information sources play a pivotal role in the breast cancer beliefs and behavioural practices towards breast self-examination among women in Lagos state, Nigeria. It is therefore recommended that a national communication policy that will incorporate the use of information sources to strategically influence the beliefs and behavioural practices of women towards breast cancer management in Nigeria be developed.

## 1. Introduction

The burden of breast cancer is high with a colossal effect on lives, families and communities. The disease has been a leading cause of death among women globally. The World Health Organisation has estimated that 2.3 million women have been infected globally, and there is a 650,000 mortality rate [1]. Though incidence in the African region was lower than in other continents except for Asia, its age-standardised mortality rate has been ranked the highest worldwide, with Nigeria having the highest mortality rate [2].

Although studies on the degree of knowledge and awareness of breast cancer in the late 1980s and early 1990s found that most women are unaware of the condition, particularly its risk factors and treatment options [3], recent studies have revealed a rise in breast cancer awareness behavioural practices, particularly among women in more industrialised cultures [4]. The same cannot be accurate for women in developing nations, particularly those in Africa’s Sub-Saharan area, where behavioural practices towards breast cancer screening are still poor [5].

For instance, studies have revealed that most Nigerian women in rural and urban regions have little or no understanding of the disease’s risk factors, symptoms and good behavioural practices [6,7]. Such a behavioural practice gap has been associated with health communication and literacy.

In the field of health communication, information sources are important for health promotion and education. Scholars say that information sources are important for raising public health awareness because they are the basis for health communication activities [8,9]. Personal and community health improvement attitudes and knowledge are positively influenced by information sources. Making well-informed decisions, therefore, requires effective health communication.

The importance of information acquisition in health behaviour promotion has been highlighted in studies on health communication [5]. The fact that breast cancer is a non-infectious invasive disease has increased the demand for communication-based treatments since a poor understanding of the disease can have various negative consequences, such as the spread of fear and the rejection of behavioural interventions [5,7]. As a result, the spread of breast cancer screening information is crucial to breast self-examination. Previous screening research has found that various sources of health information have both affirmative and negative effects on screening habits [10]. Ascertaining the most valuable sources of information for raising women’s desire to engage in breast self-examination is critical to the success of breast cancer screening and behavioural interventions.

Existing research, particularly for communication on health, has shown that communication elements may not directly impact the public [11,12]. However, much health behaviour management research has concentrated on the indirect pathways via which communication features influence public behaviour. Scholars have underlined the need to determine indirect paths through psychological difficulties. As a result, this research aims to explore how women’s acquisition of breast cancer information from various sources influences their health beliefs and behavioural practice intentions via psychological factors.

The health belief model is one of the most often used models for studying the factors of people’s intentions to adopt a behavioural practice [6,7]. It focuses on psychological aspects such as vulnerability, severity barriers, cues to action, benefits and self-efficacy. However, few researchers have examined the elements influencing one’s health attitudes. This study aimed to fill that gap by investigating women’s health communication and behaviour towards breast cancer screening in Lagos, Nigeria. The HBM was used as the theoretical foundation for this research to improve women’s health communication and behaviour towards breast cancer screening in Lagos, Nigeria. The choice of Lagos is predicated on a 2009–2016 report of Nigeria National System of Cancer Registries which ranks Lagos as the second state with the most prevalent cases of breast cancer among women in Nigeria. The analysis will be guided by the following formulated hypotheses:

**H1.**  *There is a significant relationship between information sources and women’s perceived susceptibility to breast cancer and adherence to breast self-examination*.

**H2.**  *There is a significant relationship between information sources and women’s perceived severity of breast cancer and adoption of (breast cancer) breast self-examination*.

**H3.**  *There is a significant relationship between information sources and the perceived barrier of women towards breast self-examination*.

## 2. Materials and Methods

### 2.1. Research Design

Correlational research design was employed in assessing the relationship between information sources and behavioural practices of women in Lagos, Nigeria without any form of manipulation. This helps in reflecting the degree and strength of relationships between information sources and behavioural practices of women. The results of this study were analysed in a descriptive and explanatory way using a quantitative research method called a survey. This was performed in assessing the influence of information sources on behavioural practices of women in Lagos, Nigeria.

This method is crucial because it ensures accurate analysis, interpretation, evaluation and classification of trends and connections relevant to breast cancer in the population under study.

The population of this study included women in Lagos state, southwest Nigeria. Lagos is the second most populous state in Nigeria and is described as the commercial hub of the country [13,14]. The women population of Lagos has been estimated to be 5,295,476 (National Population Commission, 2006). The choice of Lagos was predicated on the 2009–2016 report of the Nigeria National System of Cancer Registries, which ranks Lagos as the second state with the most prevalent cases of breast cancer in Nigeria. Furthermore, Fapohunda, et al. [15] observed that breast cancer is the most common cancer type among women. Similarly, the choice of Lagos is premised on the increased number of advocacy campaigns for eradicating breast cancer. Such campaigns (interpersonal, mass media and social media) have been carried out in Lagos by government and non-governmental organisations such as Run For Cure Africa Breast Cancer Foundation [16], Innovating Health & Cancer Care Foundation [17] and civil Society for Cancer Eradication in Nigeria (CISCANEN) [18] during World Breast Cancer Day on the 19th of October every year.

### 2.2. Sample Size and Sampling Techniques

Due to the demographic makeup of the study population, the qualitative method of survey was used. The researcher randomly selected a total of 400 women respondents in the age category of 18 and above, representing the semi-urban and the urban segments of Lagos state. The choice of this age category was based on the recommendations of different studies that women in their early twenties should begin breast self-examination [19,20]. The sample size of 400 respondents was premised on [21] proposition of 384 sample size for a population that is more than 1,000,000 people at a 95% confidence level and 5% sampling error. Therefore, the sampling size of the study (400) is above the proposition of 384, as proposed by [21]. The researcher recruited two trained research assistants from the two senatorial districts under investigation in Lagos state, Nigeria. The research assistants were trained to interpret for some of the respondents that did not understand English language in their mother’s tongue.

For this cross-sectional study, the large population of Lagos state was delimited to a manageable size using a multistage sampling technique. According to the submission of [22], multistage sampling technique is effective when the population is scattered over a heterogeneous area. This technique involves segmenting units into sub-populations, usually referred to as strata, within each stratum, using a hierarchical system of units [23,24].

In the first selection stage, the simple random technique was employed in selecting two out of the three senatorial districts in Lagos state (Lagos-East and Lagos-West). Furthermore, in the second stage, the simple random technique was utilised in selecting two local governments, each from the senatorial districts, making a total of four local governments (Alimosho Local Government Area, Oshodi/Isolo Local Government Area and Ikorodu, Epe). The third stage involved using the simple random technique to select two wards each from the identified local government, totaling eight wards.

At the fourth selection stage, the simple random technique was used in selecting two streets each from the wards, making it a total of sixteen streets. In the last stage, the street was stratified into residential houses. The researcher, therefore, used a systematic sampling technique in selecting residential houses that falls within the sample. To perform this, the researcher used systematic technique matrix developed by Wimmer and Dominick [25].

### 2.3. Data Analysis Procedures

Univariate was used to present the respondents selected demographic characteristics. To determine the significant relationships between information sources and behavioural practice of women towards breast self-examination, the SPSS 23 was used for the data coding while the structural equation modelling (Smart PLS 3.0) statistical tool was used to analyse the data to determine the significant relationships between information sources and behavioural practice of women towards breast self-examination. The hypothesis test was used to determine whether there is statistical proof to substantiate or nullify the proposed hypotheses. The null form was used to express all of the hypotheses tested in this study.

### 2.4. Method of Data Collection

The study employed a questionnaire in gathering data from the women respondents. The copies of questionnaire were self-administered. The researcher with the help of a research assistant ensured the questionnaires were appropriately filled. The questionnaires were translated into Pidgin and Yoruba languages of the selected study area and were translated back to English by different persons to ensure that the English version represented the actual meaning in English and Yoruba. This method helped to eliminate issues of misconception and ambiguity.

### 2.5. Ethical Clearance

Ethical clearance with the protocol number **CHREC/132/2022** to conduct the study was obtained from the Covenant University Research and Ethics Committee. Written informed consent form was given to each respondent to confirm their voluntary participation in the study. The entire research procedure was explained to the respondents with the option of withdrawal if they were no longer comfortable. Data anonymity and security were maintained, as data collected were anonymised, entered and kept in security—a protected electronic platform.

## 3. Results

The demographic profile of the respondents is depicted in Table 1. The findings show that 52.5% of the total respondents were within the ages of 20 and 29 years, while 23.3% were within the age group of 30 and 39 years. Similarly, 14.8% were within the age bracket of 40 and 49 years, 7.3% were between the ages of 50 and 59 years, while 2.3% were 60 years and above. In addition, the respondents’ marital status was revealed. The result shows that 48.8% of the total respondents were single, 47.0% were married, 1.8% were divorced and 2.5% of the study population were widows. This indicates that women, whether single or married, comprise most of the population, accounting for 95.8% of the total.

Similarly, the researchers examined the educational status of the 400 respondents, discovering that only 3.8% had no formal education, 3.3% were students, 21.0% of the study population were secondary school graduates and 72.1% were tertiary institution graduates. The bulk of the respondents, however, had finished tertiary education. This suggests that most of the respondents were educated, and their information may be trusted.

In addition, 30.5% of the 400 total respondents were students, 12.3% were artisan/handwork, 24.8% were businesswomen and the remaining 32.5% were employed. Most respondents were employed. Meanwhile, 286 respondents, representing 71.5%, identified themselves as Christians, whereas 101 respondents, representing 25.3%, identified themselves as Muslims. Traditional accounted for 2.5% of the respondents, while other religions accounted for 30.8%. Most of the respondents were Christian, accounting for 71.5% of the study population.

**H1.**  *There is a significant relationship between information sources and women’s perceived susceptibility to breast cancer and adherence to breast self-examination*.

**Figure 1:**
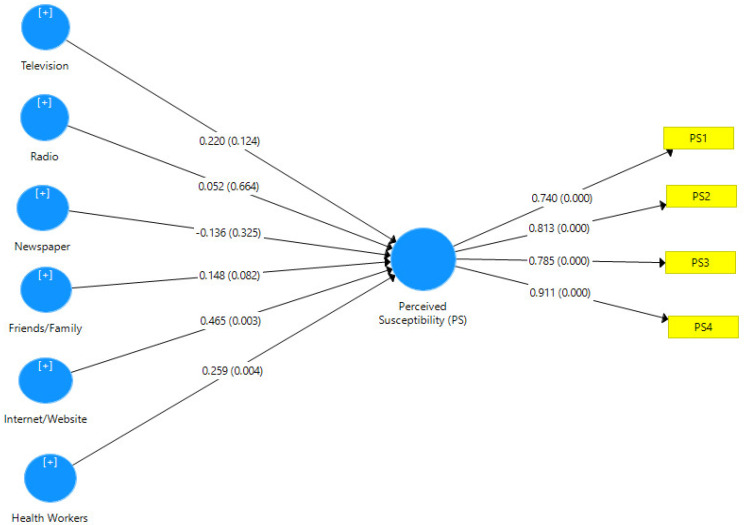
Information sources and women’s perceived susceptibility to breast cancer model.

Hypothesis one tested the relationship between information sources and women’s perceived susceptibility to breast cancer and adherence to breast self-examination. Path coefficients, t-statistics, R-square values and *p*-values were used to interpret the results. The path coefficient, as shown in Figure 1, determines the degree and strength of the correlation between the observed variables. The R-square, on the other hand, determined the amount of variance in the women’s perceived susceptibility to breast cancer and adherence to breast self-examination, as indicated by the information sources. As shown in Table 2, the *p*-value denotes the degree of probability that must be less than 0.05 to be considered significant, whereas the t-statistics denote the measured differences in standard error units.

Figure 1 shows the PLS algorithm model of information sources and women’s perceived susceptibility to breast cancer with the loading values of each item of measurement for both information sources (television, radio, newspaper, friends/family, Internet, and health workers) and women’s perceived susceptibility to breast cancer. Figure 1 also depicts the PLS Bootstrapping Model with β and P-coefficients of the value of both variables. The *p*-value determines the amount of probability.

Table 2 shows the factor loadings of all the measurement items for information sources and perceived susceptibility to breast cancer. Composite reliability, average variance extracted (AVE) computation and Cronbach Alpha were used to assess the instrument’s validity and reliability. Meanwhile, the factor loading, composite reliability, AVE and Cronbach Alpha criteria were met. Convergent and discriminant validity were also considered in the study when determining construct validity. Convergent validity is evidence of a link between information sources and perceived susceptibility to breast cancer.

The discriminant validity of information sources and perceived susceptibility to breast cancer is depicted in Table 3. The heterotrait–monotrait (HTMT) ratio of correlations method was used to assess the discriminant validity. The analysis results show that all the values are less than the HTMT 0.85 critical value. These things considered, the average heterotrait–heteromethod correlation is lower than the average monotrait–heteromethod correlation. As a result, the discriminant validity is established. Similarly, the common method bias was tested via VIF, as depicted in Table 2. The results obtained were within the recommended threshold of 3.3.

Table 4 shows the model fit for information sources and perceived susceptibility to breast cancer. All the model fit indicators were found to be acceptable. The standardised residual average between the observed matrix and the hypothesised covariance matrices is represented by SRMR. The result of SRMR is less than the 0.08 threshold point; therefore, the SRMR value of 0.071 is considered reliable, indicating a good fit.

To determine the PLS-SEM predictive relevance of the measurement constructs and the data points of indicators, the Q^2^ values were used. The Q^2^ value is 0.390, which is larger than zero. This suggests that the PLS path model has predictive relevance for the constructs. The F-square was used to determine the effect size in the same vein. The F-square is 2.117, as indicated in Table 5. This implies that the sample effect is considered large.

Table 5 depicts the smart partial least squared statistical results of hypothesis one, which focused on the relationship between information sources and perceived susceptibility to breast cancer. Generally, the findings show that information sources have a significant effect on perceived susceptibility to breast self-examination at (β = 0.803, R^2^ = 0.644, t-statistics = 12.763 > 1.96, *p*-value =0.000 < 0.05). The Path coefficient of 0.803 implied a high degree of relationship between information sources and perceived susceptibility to breast cancer. The R^2^ value of 0.644 indicates that information sources can explain a 64.4% variance in women’s perceived susceptibility to breast cancer.

Specifically, out of the six information sources considered in this study, it was discovered that the Internet has the most predictive value at the standardised coefficient value of 0.465, followed closely by health workers in the prediction of perceived susceptibility to breast cancer. However, television, radio, newspaper and friends/family do not contribute significantly to the model.

**H2.**  *There is a significant relationship between information sources and women’s perceived severity of breast cancer and adoption of (breast cancer) breast self-examination*.

**Figure 2:**
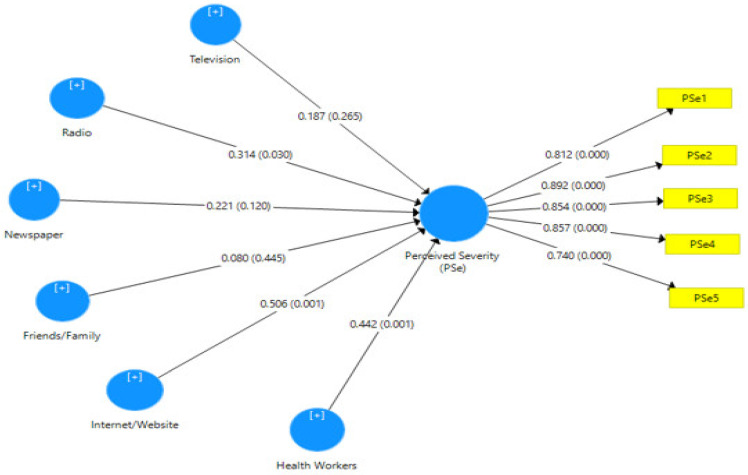
Information sources and women’s perceived severity of breast cancer model.

Hypothesis two tested the relationship between information sources and women’s perceived severity of breast cancer and adherence to breast self-examination. Path coefficients, t-statistics, R-square values and *p*-values were used to interpret the results. As shown in Figure 2, the path coefficient determines the degree and strength of the correlation between the observed variables. The R-square, on the other hand, determines the amount of variance in the women’s perceived severity of breast cancer and adoption of (breast cancer) breast self-examination, as indicated by the information sources. As shown in Table 6, the *p*-value denotes the degree of probability that must be less than 0.05 to be considered significant.

Figure 2 shows the PLS algorithm model of information sources and women’s perceived severity of breast cancer with the loading values of each item of measurement for both information sources (television, radio, newspaper, friends/family, Internet and health workers) and women’s perceived severity to breast cancer. Figure 2 also depicts the PLS Bootstrapping Model with β and P-coefficients of the value of both variables. The *p*-value determines the amount of probability.

Table 6 shows the factor loadings of all the measurement items for the perceived severity of breast cancer. Composite reliability, average variance extracted (AVE) computation and Cronbach Alpha were used to assess the instrument’s validity and reliability. Meanwhile, the factor loading, composite reliability, AVE and Cronbach Alpha criteria were met. Convergent and discriminant validity were also taken into account in the study when determining construct validity. Convergent validity is evidence of a link between information sources and the perceived severity of breast cancer examination.

The discriminant validity of information sources and perceived severity of breast cancer examination is depicted in Table 7. The heterotrait–monotrait (HTMT) ratio of correlations method was used to assess the discriminant validity. The analysis results show that all the values are less than the HTMT 0.85 critical value. These things considered, the average heterotrait–heteromethod correlation is lower than the average monotrait–heteromethod correlation. As a result, the discriminant validity is established. Similarly, the common method bias was tested via VIF, as depicted in Table 6. The results obtained were within the recommended threshold of 3.3. This can be concluded that the model is free from common method bias.

Table 8 displays the model fit for information sources and the perceived severity of breast cancer. All the model fit indicators were found to be acceptable. The standardised residual average between the observed matrix and the hypothesised covariance matrices is represented by SRMR. The result of SRMR is less than the 0.08 threshold point; therefore, the SRMR value of 0.069 is considered reliable, indicating a good fit.

To determine the PLS-SEM predictive relevance of the constructs of measurement and the data points of indicators, the Q^2^ values were used. The Q^2^ value is 0.329, which is larger than zero. This suggests that the PLS path model has predictive relevance for the constructs. In the same vein, the F-square was used to determine the effect size. The F-square is 2.210, as indicated in Table 9. This implies that the sample effect is considered large.

Table 9 depicts the smart partial least squared statistical results of hypothesis two, which focused on the relationship between information sources and the perceived severity of breast cancer. Generally, the findings show that information sources have a significant effect on perceived susceptibility to breast cancer at (β = 0.743, R^2^ = 0.553, t-statistics = 2.662 > 1.96, *p*-value = 0.000 < 0.05). The path coefficient of 0.743 implies a high degree of relationship between information sources and the perceived severity of breast cancer. The R^2^ value of 0.553 indicates that information sources can explain a 55.5% variance in women’s perceived severity of breast cancer.

Specifically, out of the six information sources considered in this study, it was discovered that the Internet has the most predictive value at the standardised coefficient value of 0.506, followed closely by health workers and radio information sources at 0.422 and 0.316, respectively, in the prediction of perceived severity to breast cancer. However, television, newspapers and friends/family do not contribute significantly to the model.

**H3.**  *There is a significant relationship between information sources and the perceived barrier of women towards breast self-examination*.

**Figure 3:**
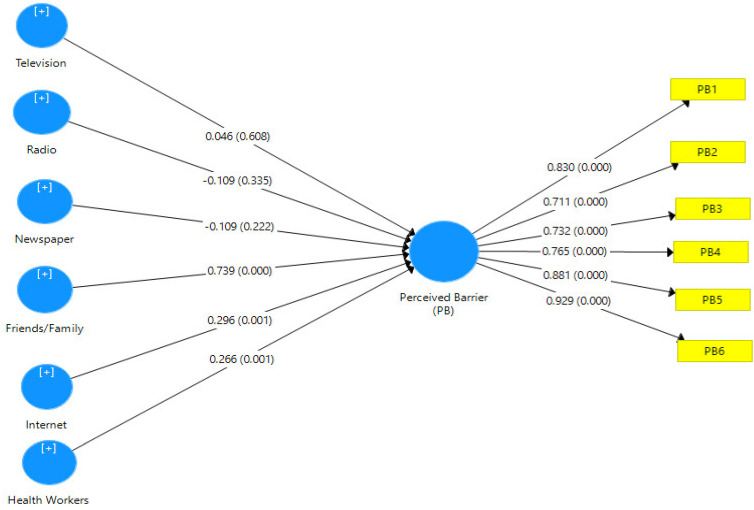
Information sources and perceived barriers of women towards breast self-examination model.

Hypothesis three tested the relationship between information sources and the perceived barrier of women towards breast self-examination. Path coefficients, t-statistics, R-square values and *p*-values were used to interpret the results. The path coefficient, as shown in Figure 3, determines the degree and strength of the correlation between the observed variables. The R-square, on the other hand, determines the amount of variance in the perceived barrier of women towards breast self-examination, as indicated by the information sources. As shown in Table 10, the *p*-value denotes the degree of probability that must be less than 0.05 to be considered significant.

Figure 3 shows the PLS algorithm model of information sources and perceived barriers of women towards breast self-examination with the loading values of each item of measurement for both information sources (television, radio, newspaper, friends/family, Internet and health workers) and perceived barrier of women towards breast self-examination. Figure 3 also depicts the PLS Bootstrapping Model with β and P-coefficients of the value of both variables. The *p*-value determines the amount of probability.

Table 10 displays the factor loadings of all the measurement items for the perceived barrier of women towards breast self-examination. Composite reliability, average variance extracted (AVE) computation and Cronbach Alpha were used to assess the instrument’s validity and reliability. Meanwhile, the factor loading, composite reliability, AVE and Cronbach Alpha criteria were met. Convergent and discriminant validity were also taken into account in the study when determining construct validity. Convergent validity is evidence of a link between information sources and the perceived barrier of women towards breast self-examination.

The discriminant validity of information sources and perceived barrier of women towards breast self-examination is depicted in Table 11. The heterotrait–monotrait (HTMT) ratio of correlations method was used to assess the discriminant validity. The results of the analysis show that all the values are less than the HTMT 0.85 critical value. These things considered, the average heterotrait–heteromethod correlation is lower than the average monotrait–heteromethod correlation. As a result, the discriminant validity is established. Similarly, the common method bias was tested via VIF, as depicted in Table 10. The results obtained were within the recommended threshold of 3.3. This can be concluded that the model is free from common method bias.

Table 12 displays the model fit for information sources and the perceived barrier of women towards breast self-examination. All the model fit indicators were found to be acceptable. The standardised residual average between the observed matrix and the hypothesised covariance matrices is represented by SRMR. The result of SRMR is less than the 0.08 threshold point; therefore, the SRMR value of 0.079 is considered reliable, indicating a good fit.

Additionally, to determine the PLS-SEM predictive relevance of the constructs of measurement and the data points of indicators, the Q^2^ values were used. The Q^2^ value is 0.497, which is larger than zero. This suggests that the PLS path model has predictive relevance for the constructs. In the same vein, the F-square was used to determine the effect size. The F-square is 3.110, as indicated in Table 13. This implies that the sample effect is considered large.

Table 13 depicts the smart partial least squared statistical results of hypothesis three, which focused on the relationship between information sources and the perceived barrier of women towards breast self-examination. Generally, the findings show that information sources have a significant effect on perceived susceptibility to breast cancer at (β = 0.764, R^2^ = 0.583, t-statistics = 11.523 > 1.96, *p*-value = 0.000 < 0.05). The path coefficient of 0.764 implies a high degree of relationship between information sources and the perceived barrier of women towards breast self-examination. The R^2^ value of 0.583 indicates that information sources can explain a 58.3% variance in the perceived barrier of women towards breast self-examination.

Specifically, out of the six information sources considered in this study, it was discovered that friends and family have the most predictive value at the standardised coefficient value of 0.739, followed closely by Internet and health workers’ information sources at 0.296 and 0.266, respectively, in the prediction of the perceived barrier of women towards breast self-examination. However, television, newspapers and radio do not contribute significantly to the model.

## 4. Discussion

Although previous studies concerning perceived susceptibility of breast self-examination globally and in Nigeria have majorly centered on a health context, ignoring the role played by the information sources by Abhang and Lopez [26], few others have examined information sources in relationship to women’s perceived susceptibility to breast cancer.

The test of hypothesis one focused on the possibility of information sources (television, radio, newspaper, family/friends, Internet and health workers) significantly affecting women’s perceived susceptibility to breast cancer and adherence to breast self-examination. The concepts of information sources and perceived susceptibility have long been a subject of research among scholars [27,28]. Out of all the information sources examined in this study, only the Internet/websites and health workers significantly influenced women’s perceived susceptibility to breast cancer. Additionally, the coefficient table, model fit, discriminant validity and construct validity and reliability indicated that information sources used to access woman’s perceived susceptibility towards adherence to BSE, which are: the Internet and health workers, all have a significant effect on how women believe in being diagnosed with breast cancer. This could be attributed to the need for these information sources by Lagos women to access a wide variety of information relating to adherence to breast self-examination. The Internet’s significance can be because of younger women relying more on the Internet for information sources, as they are Internet- and tech-savvy [29]. In addition, there is an increased use of smartphones with Internet access capabilities of health information sources due to easy access and everyday use, which has resulted in a high degree of human beings’ believability [30].

This result is similar to earlier research by Jackson et al. [31]; Ayandipo et al. [32] that identified health workers as the primary sources of health information among cancer patients. This conclusion also corroborates the findings of a systematic review and empirical research on information sources among cancer patients, which indicated that health professionals were the most often used source of information [33,34].

Since healthcare professionals play significant roles in the lives of women with breast cancer, it is not surprising that they were the primary source of information. However, some studies revealed that personal communication with some healthcare professionals revealed that breast cancer patients are primarily given verbal information because most have low literacy levels [35,36].

The perceived susceptibility is a significant determinant and foundation for attitudinal change [37,38]. In a study carried out by Masoumi [39] to find out if perceived susceptibility mediates attitudinal change and behaviour, it was discovered that susceptibility is predicated on a significant perception of the risk of acquiring an illness or disease. Information sources on the Internet and from health workers invariably influence women’s susceptibility level and engender attitudinal change towards BSE.

Hypothesis two tested if there is a significant relationship between information sources and the perceived barrier of women towards breast self-examination. While many respondents reported hearing about breast cancer from traditional sources such as radio, television and newspapers, as well as interpersonal sources such as health professionals, friends and family, most respondents cited the family as their source.

Because it is human nature for people to confide in their closest circle about such delicate/sensitive material, family and friends could be viewed as having a significant impact on their attitudes. Since respondents believe that friends may provide various services, including material and emotional support, they are a good source of aid and a booster for women’s attitudes about learning more about the disease. However, Alberti et al. [40] stress that health literacy may influence the effectiveness of family health communication. Inadequate health literacy could inevitably result in information distortion via this channel.

The Internet/websites, on the other hand, have had a significant impact on women’s views around breast cancer because it has increased their awareness, competence and participation in health decision making. Additionally, independent Internet inquiries can supplement and synergize often time-constrained doctor–patient interactions in the clinic [41]. The Internet has been identified as a vital source of health information [42]. Due to its similarities to traditional media in its ability to reach a broad audience, the Internet may economically and geographically reach a wide populace. Facebook, Instagram, Twitter, WhatsApp and Snapchat are typically employed for this purpose. According to Isa Ali Ibrahim, Nigeria’s Minister of Communication, 75% of Nigerians with Internet access use social media [43].

These findings indicate that Internet users generally view cancer-related material as valuable and that the majority addressed Internet-derived information with their healthcare providers and thought that clinicians listened to such information. Nevertheless, some of these respondents exhibited skepticism regarding the dependability of Internet-based medical information. Not only did the Internet/website encourage respondents to seek health information quickly, but it also encouraged distrust in their thinking. Not to mention a sensitive aspect of people’s lives—their health—the trustworthiness and veracity of online information have frequently been questioned. This has altered their views towards examining the information they obtain on the Internet and cross-checking it with other sources, such as family/friends, health professionals, etc.

Even though interpersonal communication approaches to breast cancer communication take longer and can only reach a limited number of people compared to mainstream media, some of the respondents in this study have accessed enough information on breast self-examination through interpersonal communication channels. The study reveals that healthcare professionals are considered the source of knowledge among other interpersonal communication channels.

## 5. Conclusions

This study concludes that most of the respondents seek information on breast self-examination from various sources to some extent. The study demonstrates that a higher percentage of women often seek information about breast self-examination from various sources, as they also often read/watch or hear information about breast cancer from various sources. This research further revealed that Internet communication channels predominantly influenced the perceived susceptibility and severity of women in Lagos state towards breast self-examination. Similarly, interpersonal communication engendered the barrier perception of study participants towards the practice of BSE. This study has also confirmed the postulation of the health belief model, which states that the adaptation of positive health behaviour depends on an individual’s vulnerability and severity perception.

## Figures and Tables

**Table 1:** Demographic Characteristics.

	Frequency	Percentage
**Age**		
20–29	210	52.5
30–39	93	23.3
40–49	59	14.8
50–59	29	7.3
60-above	9	2.3
**Total**	**400**	**100%**
**Marital Status**		
Single	195	48.8
Married	188	47.0
Divorced	7	1.8
Widows	10	2.5
**Total**	**400**	**100%**
**Level of Education**		
Uneducated	15	3.8
Student	13	3.3
Secondary	84	21.0
Tertiary	288	72.1
**Total**	**400**	**100%**
**Occupation**		
Student	122	30.5
Artisan/handwork	49	12.3
Businesswoman	99	24.8
Employed	130	32.5
**Total**	**400**	**100%**
**Religion**		
Christianity	286	71.5
Islam	101	25.3
Traditional	10	2.5
Others	3	0.8
**Total**	**400**	**100%**

**Table 2:** Construct validity and reliability for Hypothesis One.

	Loading	VIF	t-Statistics	*p* Value	AVE	Composite Reliability	Cronbach’s Alpha
Constructs	**≥0.7**	**<3.0**	**>1.96**	**<0.05**	**≥0.5**	**≥0.8**	**>0.7**
**Information Sources**					**0.656**	**0.809**	**0.758**
Television	0.782	2.487	1.512	0.082			
Radio	0.789	2.960	0.671	0.664			
Newspaper	0.796	3.001	1.129	0.322			
Friends/Family	0.799	2.782	1.576	0.082			
Internet	0.884	1.741	9.040	0.003			
Health Workers	0.805	2.023	5.012	0.004			
**Perceived Susceptibility (PS)**					**0.664**	**0.887**	**0.829**
PS1	0.740	1.813	11.277	0.000			
PS2	0.813	1.222	21.618	0.000			
PS3	0.785	1.941	16.665	0.000			
PS4	0.911	1.335	48.011	0.000			

**Table 3:** Discriminant validity.

	Friends	Health Workers	Internet	Newspaper	PS	Radio	Television
Friends							
Health Workers	0.299						
Internet	0.450	0.306					
Newspaper	0.417	0.717	0.521				
**PS**	0.522	0.459	0.823	0.515			
Radio	0.187	0.311	0.595	0.520	0.552		
Television	0.441	0.276	0.807	0.493	0.759	0.626	

**Table 4:** Model fit.

	Estimated
SRMR	0.071
d_ULS	0.625
d_G	0.280
Chi-Square	140.351
NFI	0.947

**Table 5:** Coefficient value of Hypothesis One.

Variables	Path Coefficient	SE	T-Statistics	*p*-Values	R^2^	F^2^	Q^2^	Decision
**Information Sources → PS**	**0.803**		**12.763**	**0.005**	**0.644**	**2.117**	**0.390**	**Significant**
Friends/Family → PS	0.148	0.085	1.739	0.082	0.021	0.040		Insignificant
Health Worker → PS	0.257	0.090	2.897	0.004	0.066	0.183		Significant
Internet → PS	0.465	0.156	2.990	0.003	0.216	0.188		Significant
Newspaper → PS	0.136	0.138	0.985	0.325	0.018	0.017		Insignificant
Radio → PS	0.052	0.121	0.435	0.664	0.003	0.004		Insignificant
Television → PS	0.220	0.143	1.537	0.124	0.048	0.042		Insignificant

**Table 6:** Construct validity and reliability for Hypothesis Two.

	Loading	VIF	*p*- Value	AVE	Composite Reliability	Cronbach’s Alpha
**Constructs**	**≥0.7**	**<3.0**	**<0.05**	**≥0.5**	**≥0.8**	**>0.7**
Perceived Severity (PSe)				0.693	0.918	0.889
PSe1	0.812	2.006	0.000			
PSe2	0.892	2.321	0.000			
PSe3	0.854	1.884	0.000			
PSe4	0.859	2.115	0.000			
PSe5	0.740	1.515	0.000			

**Table 7:** Discriminant validity.

	Friends	Health Workers	Internet	Newspaper	PSA	Radio	Television
Friends							
Health Workers	0.299						
Internet	0.450	0.306					
Newspaper	0.189	0.311	0.595				
PSe	0.417	0.658	0.209	0.355			
Radio	0.441	0.717	0.521	0.520	0.623		
Television	0.441	0.276	0.807	0.626	0.239	0.493	

**Table 8:** Model fit.

	Estimated
SRMR	0.069
d_ULS	0.680
d_G	0.301
Chi-Square	133.785
NFI	0.939

**Table 9:** Coefficient value of Hypothesis Two.

Variables	Path Coefficient	SE	T-Statistics	*p*-Values	R^2^	F^2^	Q^2^	Decision
**Information Sources** **→ PS**	**0.743**		**2.662**	**0.002**	**0.553**	**2.210**	**0.329**	**Significant**
Friends/Family → PS	0.080	0.105	0.764	0.445	0.006	0.009		Insignificant
Health Worker → PS	0.442	0.131	3.371	0.001	0.195	0.204		Significant
Internet → PS	0.506	0.157	3.221	0.001	0.256	0.172		Significant
Newspaper → PS	0.221	0.142	1.554	0.120	0.049	0.054		Insignificant
Radio → PS	0.316	0.144	2.176	0.030	0.100	0.072		Significant
Television → PS	0.187	0.168	1.114	0.265	0.035	0.024		Insignificant

**Table 10:** Construct validity and reliability for Hypothesis Three.

	Loading	VIF	*p*- Value	AVE	Composite Reliability	Cronbach’s Alpha
Constructs	**≥0.7**	**<3.0**	**<0.05**	**≥0.5**	**≥0.8**	**>0.7**
**Perceived Barrier (PB)**				0.659	0.920	0.894
PB1	0.830	1.774	0.000			
PB2	0.711	2.666	0.000			
PB3	0.732	1.901	0.000			
PB4	0.765	2.330	0.000			
PB5	0.881	1.781	0.000			
PB6	0.929	1.800	0.000			

**Table 11:** Discriminant validity.

	Friends/ Family	Health Workers	Internet	Newspaper	PB	Radio	Television
Friends/Family							
Health Workers	0.299						
Internet	0.311	0.187					
Newspaper	0.421	0.555	0.325				
PB	0.814	0.387	0.528	0.428			
Radio	0.306	0.499	0.595	0.628	0.410		
Television	0.276	0.441	0.626	0.527	0.422	0.807	

**Table 12:** Model fit.

	Estimated
SRMR	0.079
d_ULS	0.622
d_G	0.311
Chi-Square	161.595
NFI	0.925

**Table 13:** Coefficient value of Hypothesis Three.

Variables	Path Coefficient	SE	T-Statistics	*p*-Values	R^2^	F^2^	Q^2^	Decision
**Information Sources** **→** **PS**	**0.764**		**11.523**	**0.001**	**0.583**	**3.110**	**0.497**	**Significant**
Friends/Family → PS	0.739	0.052	14.346	0.000	0.546	1.988		Significant
Health Worker → PS	0.266	0.080	3.351	0.001	0.071	0.206		Significant
Internet → PS	0.296	0.086	3.455	0.001	0.089	0.221		Significant
Newspaper → PS	0.109	0.089	1.222	0.222	0.012	0.026		Insignificant
Radio → PS	0.109	0.113	0.964	0.335	0.012	0.015		Insignificant
Television → PS	0.046	0.090	0.513	0.608	0.002	0.003		Insignificant

## Data Availability

Data will be made available on request.
